# Prevalence and diversity of *Babesia* spp. in questing *Ixodes ricinus* ticks from Norway

**DOI:** 10.1186/1756-3305-5-156

**Published:** 2012-08-04

**Authors:** Øivind Øines, Jana Radzijevskaja, Algimantas Paulauskas, Olav Rosef

**Affiliations:** 1 Norwegian Veterinary Institute, P.O. Box 750, Sentrum, 0106, Oslo, Norway; 2 Department of Biology, Vytautas Magnus University, Vileikos str. 8, Kaunas, LT-44404, Lithuania; 3 ATP-Innovation AS, 3800, Bø i Telemark, Norway

**Keywords:** *Babesia* spp, Questing *Ixodes ricinus*, Zoonosis, Piroplasmosis, Realtime PCR, Prevalence, Sequencing

## Abstract

**Background:**

*Ixodes ricinus* ticks transmit *Babesia* species to vertebrate hosts. Using molecular tools we were able to detect the presence of this piroplasmid in its vector. The aims of this study were to investigate the prevalence and identity of *Babesia* species in questing ticks collected in various areas of Norway.

**Methods:**

DNA from questing *l. ricinus* ticks were examined with a realtime PCR for the presence of *Babesia*. Positive samples of tick DNA were identified to species using PCR, and sequence analysis.

**Results:**

From a total of 1908 questing *l. ricinus* ticks, 17 (0.9%) indicated the presence of *Babesia* spp. after realtime-PCR screening. *Ixodes ricinus* harbouring *Babesia* spp. was detected in 9 out of 22 localities. Further molecular analyses of DNA from these positive ticks indicate the presence of *Babesia venatorum*, *B. divergens*, *B. capreoli* and a currently undescribed *Babesia* in Norwegian ticks. The most prevalent was *B. venatorum* found in 71% of the positive ticks.

**Conclusions:**

A total of 17 out of 1908 (0.9%) ticks were positive for *Babesia*. Our data confirm that there are several *Babesia* species in ticks in Norway. *Babesia venatorum* was the most prevalent. This species has a zoonotic potential and may cause human babesiosis following a tick bite.

## Background

*Ixodes ricinus* is ubiquitous in Southern Norway with the highest density near the coast line [1,2]. This tick has the potential to transmit a range of zoonotic pathogens such as tick borne encephalitis virus (TBEV), bacteria causing Lyme disease and granulocytic anaplasmosis, but can also harbor blood parasites such as intraerythrocytic *Babesia* spp. The parasite can cause malaria-like syndrome in humans and animals as the red blood cell bursts during infections. A fatal *Babesia divergens* infection in 1956 was the first confirmed case of human babesiosis [3] and ever since, *B. divergens* has been regarded as a causative agent of a potentially life threatening zoonotic infection in humans [4,5].

*Babesia divergens* may cause disease in healthy and immunocompetent persons [6]. In Europe, *B. divergens* and *B. venatorum*^a^ are capable of causing severe disease in man, and most often severe cases are described from splenectomised patients [7]. However, in 2004 a fatal babesiosis case was reported in Finland where the diseased person was not splenectomized [8].

In Norway, only one human case of human babesiosis has been described in a splenectomized patient [9], but as this patient had been travelling some time before onset of the disease, it may not be regarded as an autochthonous acquired case. The main pathogen causing human babesiosis in Europe has been regarded as *B. divergens*, however, detailed molecular confirmation has not always been applied to these cases, hence correct diagnosis of the disease causing agent may not always have been carried out. Additionally, the frequent use of serology for diagnosis prevents correct identification to species level.

*Babesia* also infect and cause disease in other vertebrate hosts. In a study of pastured cows from southern Norway, immunofluorescence (IFAT) revealed that 27% of the sera investigated showed signs of *Babesia* antibodies [9]. It has been thought until recently that the only *Babesia* sp. present in Norway were the causative agent of red water disease in cattle, *B. divergens* and *B. microti* found in rodents [10]. Recently, an autochthonous canine babesiosis case was reported in a dog from Oslo [11]. Additionally, 4 ticks found on migrating birds in Norway carried *B. venatorum*[12]. Two ticks came from birds having an eastern migratory route and two came from birds that migrate to Norway via the Atlantic coast of continental Europe [12]. It was hypothesized that these infected ticks were hitchhikers from continental and/or Eastern Europe [12]. In this study we wanted to investigate the prevalence and the species of *Babesia* in host seeking *I. ricinus* ticks in various areas of Norway by using molecular tools.

## Methods

### Sampling of questing *I. ricinus* ticks

**Figure 1:**
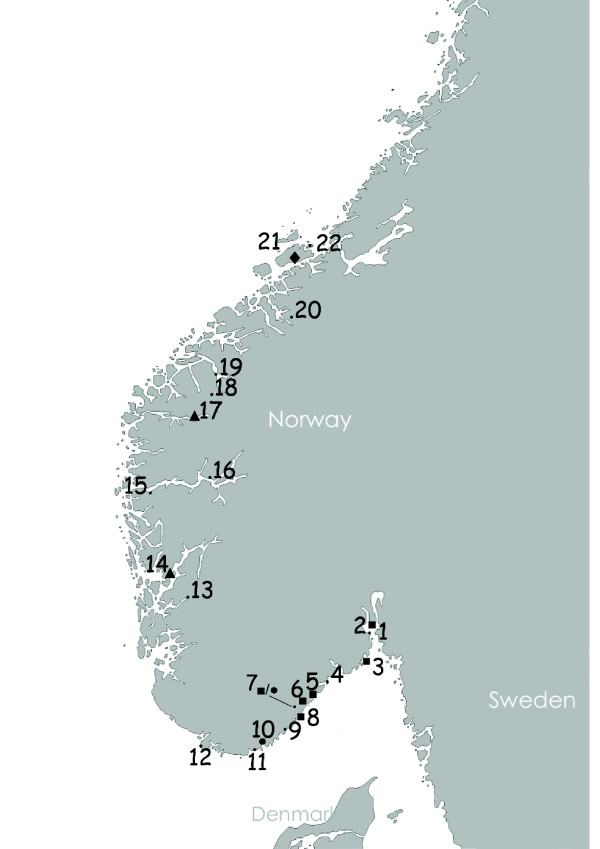
**Map of sampling localities.1)** Mølen, **2)** Løvøya, **3)** Hvasser, **4)** Jomfruland, **5)** Risør, **6)**Tvedestrand, **7)** Hinnebu, **8)** Tromøy, **9)** Tjore, **10)** Odderøya, **11)** Søgne, **12)** Lista, **13)** Etne, **14)** Mundheim, **15)** Brekke **16)** Hermansverk, **17)** Utvik, **18)** Hellesylt, **19)** Stranda, **20)** Surnadal, **21)** Tippheia, Hitra and **22)** Fjellværsøy, Hitra. Localities marked with ■ indicate positive sample(s) of *Babesia venatorum*; ▴- *Babesia divergens*, ● – *Babesia capreoli*; ♦- *Babesia CHI*-like strain.

Unfed *I. ricinus* ticks (nymphs and adults) were collected in spring-summer (April to June) seasons during 2006-2008 from 22 localities (Table 1) spread from southeast to northwest Norway (Figures 1).

**Table 1:** Prevalence of *Babesia* spp. in host seeking *Ixodes ricinus* ticks

**Map ID**	**Location**	**Year**	**No**	**Nymphs**	**Female**	**Male**
1	Mølen**	2006	50(1)	7	18(1)	25
2	Løvøya**	2006	60	14	23	23
3	Hvasser**	2006	23(1)	11	8(1)	4
4	Jomfruland	2006**	91	75	8	8
		2007	120	60	50	10
		2008	94	25	29	40
5	Risør	2006	59 (1)	22	19	18(1)
6	Tvedestrand	2006	46 (1)	19	10	17(1)
		2007	70(1)	5	35	30(1)
7	Hinnebu	2006	106(2)	32(2)	42	32
		2007	105(3)	5(1)	48 (2)	52
		2008	58(1)	0	32	26(1)
8	Tromøy	2006	41(2)	6	24(2)	11
		2007	37	5	13	19
9	Tjore	2006	24	16	4	4
		2007	48	7	19	22
		2008	52	14	24	14
10	Odderøya	2007	76(1)	0	30(1)	46
11	Søgne	2007	25	14	16	5
12	Lista	2007	60	0	28	32
13	Etne	2007	48	48	0	0
14	Mundheim	2007	46(1)	33(1)	8	5
15	Brekke	2006	9	3	2	4
16	Hermansverk	2007	48	11	33	4
17	Utvik	2007	48(1)	0	22	26(1)
18	Hellesylt	2007	9	1	5	3
19	Stranda	2007	58	29	13	16
20	Surnadal	2007	49	0	33	16
21	Tippheia, Hitra	2006	97(1)	89(1)	5	3
		2007	58	0	18	40
		2008	46	34	5	7
22	Fjellværsøy, Hitra	2006	64	56	2	6
		2007	83	60	23	0
Total in 22 locations			1908	701	639	568
Positive ticks (n)/%			(17)/0.9%	(5)/0.7%	(7)/1.1%	(5)/0.9%

*In parenthesis the number of ticks with *Babesia* spp.

** Radzijevskaja et al. 2008.

The ticks were collected by using a standard flagging method for collecting active ticks on vegetation [13]. The methods involved dragging a 1 m^2^ piece of white cotton cloth over the vegetation and checking for questing ticks every 10 m. All attached ticks were removed from the cloth and collected into sealed vials containing 70% ethanol and stored at 4°C until analyzed.

### DNA extraction

All ticks were analyzed individually. Extraction of DNA from questing (unfed) ticks was carried out by lysis in ammonium hydroxide (NH_4_OH), [14,15] with 80 μl for nymphs and 100 μl for adults. A 2.5% ammonia solution was used in a 0.5 ml microcentrifuge tube and heated at 99°C for 25 min in a thermostat block (Heating/cooling dry block, BioSan*,* England). After a brief centrifugation (in order to collect condensate from the cap and sides of the tube) the tubes were opened and heated at 99°C for approximately 10-15 min to evaporate ammonia. The lysates were stored at 4°C until use as templates for PCR or at –20°C for longer periods.

### Identification of *Ixodes ricinus*

The identification of ticks was performed by using appropriate taxonomic keys [13,16] and by molecular assays described in Radzijevskaja *et al*. [17].

### Detection and identification of *Babesia* spp

For detection, the primers BdiF (5′-CAG CTT GAC GGT AGG GTA TTG G-3′, BdiR (5′-TCG AAC CCT AAT TCC CCG TTA-3′) and TaqMan Probe BdiT (5′-6-FAM-CGA GGC AGC AAC GG-MGB-3′) were used to amplify a 62 bp fragment of the 18S rRNA gene of *Babesia* spp*.*[18]. Negative and positive controls were included in all runs. A total of 1908 (639 female, 568 male and 701 nymphs) were examined individually.

### Sequencing

**Figure 2:**
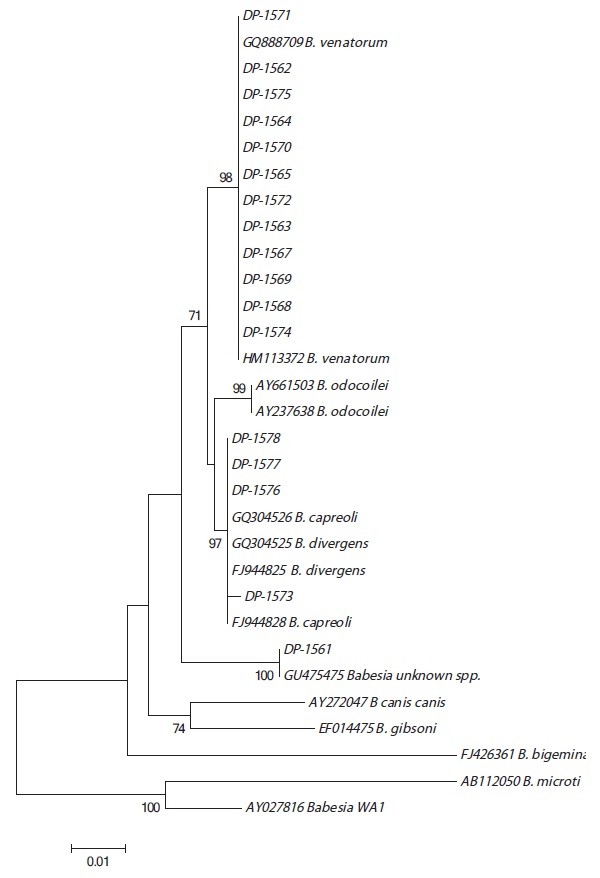
**The evolutionary relationship inferred using Neighbor-Joining method in 3′ end of 18S.** Percentages of replicate trees from the bootstrap test (500 replicates) are shown next to branches. The distances were computed using Jukes-Cantor method. The rate variation among sites was modeled with a gamma distribution (shape parameter = 1). The analysis involved a total of 31 nucleotide sequences. All positions containing gaps and missing data were eliminated from analysis. A total of 436 positions were included in the final dataset. These positions corresponded to nucleotides between positions 1076 to 1549 in the *B. divergens* sequence (FJ944825).

Ammonium hydroxide DNA extracts with tick material from samples positive from the realtime PCR screening were purified to maximize DNA yield using Qiagen DNeasy blood and tissue kit (Qiagen, Germany) after overnight lysis in buffer and Proteinase K. The DNA template was then run in a nested PCR using primers from Protocol II by Zintl *et al.*[19]. An additional PCR by the same authors (Protocol I) [19] was used for some of the samples after initial analysis of the sequences obtained from Protocol II [19]. One DNA positive control “Mia” from a bovine babesiosis case at Flekke in 2007 (20-30 km N from location 15 ‘Brekke’, Figures 1) was included in the analysis. PCR-products were visualized on a 1.5% agarose gel using GelRed™ DNA-staining reagent (Biotium, Hayward, USA). PCR products were cleaned using Macherel-Nagel Nucleospin Gel and PCR-cleanup kit. Products were subsequently sequenced on the AVANT automated sequencer (ABI) using Big Dye sequence mix and the PCR primers for the sequence reaction. Chromatograms from the automated sequencer were imported into Vector NTi Contig Express in Vector Nti Advance® 11.5 (Invitrogen, Carlsbad, USA). Chromatograms were manually edited, trimmed prior to assembly. A BLASTn search (http://blast.ncbi.nlm.nih.gov/Blast.cgi) was performed on each of the sequences produced, and top hits were imported and aligned in Align X module of Vector Nti. The alignment of the sequences from the samples and a selected group of top database matches (Figures 2) was exported into MEGA [20] and the nucleotide positions 1076-1549 relative to a sequence from *B. divergens* (FJ944825), included in the first cluster analysis. Additional analysis of products from Protocol I using positions 481 to 979 of the same Genbank molecule was carried out for the samples unidentifiable from the first analysis.

For tree construction a simple Neighbor-Joining [21] clustering was performed using Jukes-Cantor algorithm [22] and with a bootstrap analysis of 500 replicates. The results from these analyses were used to identify the samples to species level.

## Results

### Identification of *I. ricinus*

Ticks were identified to *I. ricinus* by morphological examination and by successful amplification of a 150 bp product from the 5.8s rRNA pcr [17]. No molecular subtyping was performed on the ticks in this study.

### Real-time PCR prevalence

*Babesia* spp. positives were found in 17 (0.9%) out of 1908 *I. ricinus* ticks, representing 9 out of the 22 sites investigated. The parasite was distributed in adult females with 7 out of 639 (1.1%) in adult males with 5 out of 568 (0.9%) and in nymphs with 5 out of 701 (0.7%) respectively. The highest rate was found in ticks from Hvasser with 4.3% (1 out of 23). *Babesia* spp. were found every year at Hinnebu with an average of 2.2% (6 out of 269) (Table 1). The locations where *Babesia* species were found are indicated in Figures 1.

### Nested PCR and sequencing

**Figure 3:**
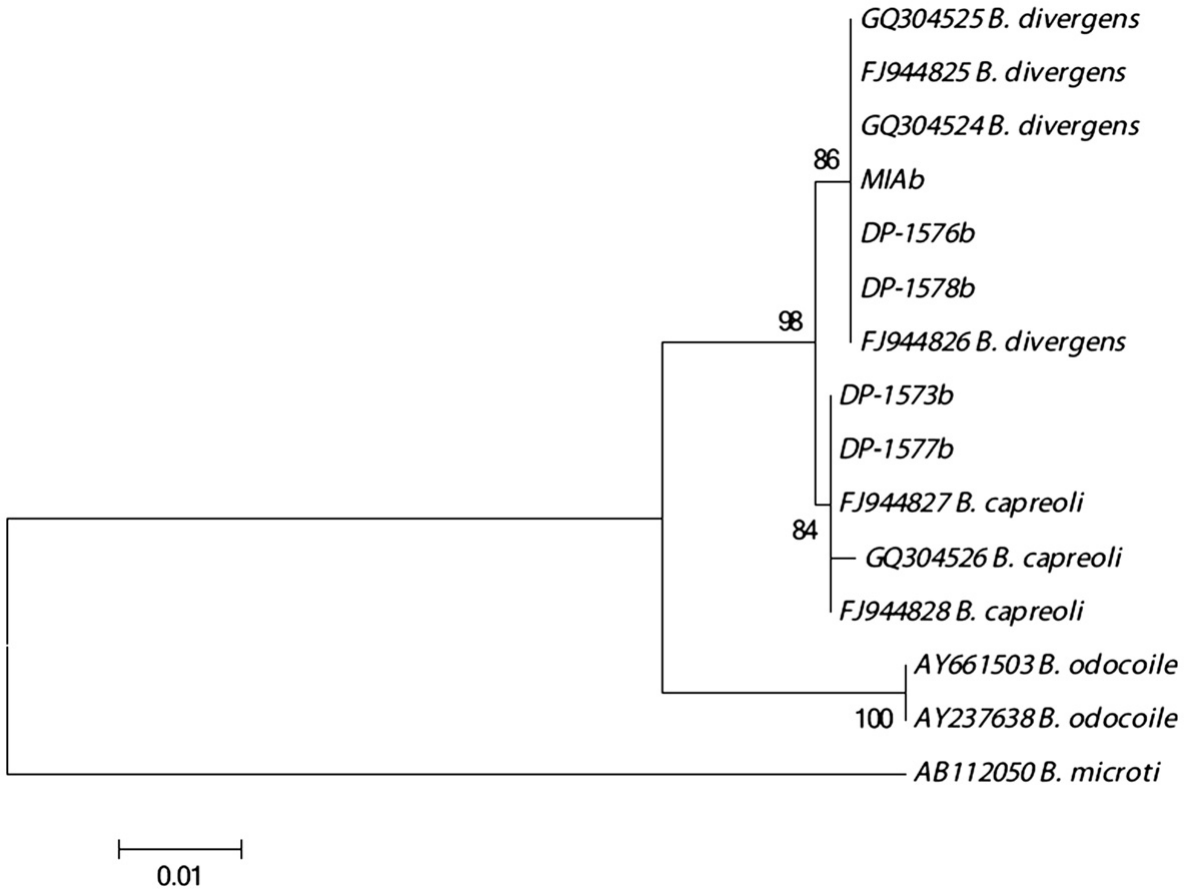
**The evolutionary relationship inferred using Neighbor-Joining method using data from 5′ end of 18S.** The evolutionary history of a total of 15 sequences was inferred using the Neighbor-Joining method. Next to branches are the percentages of bootstrap replicate trees (500 replicates) in which the samples clustered together are shown next to the branches. The evolutionary distances were computed using Jukes-Cantor and the rate variation among sites was modeled with a gamma distribution. (= 1). The analysis included 15 nucleotide sequences. All positions containing gaps and missing data were eliminated. There were a total of 498 positions in the final dataset. Nucleotides used in the calculation corresponded to the nucleotide positions from 481 to 979, relative to *B. divergens* sequence (FJ944825).

A total of 17 ticks yielded sequence products corresponding to *Babesia* spp. Sequences from twelve of them (71%) matched sequences of *B. venatorum* isolates (DP-1562 (JX042314), DP-1563 (JX042315), DP-1564 (JX042316), DP-1565 (JX042317), DP-1567 (JX042318), DP-1568 (JX042319), DP-1569 (JX042320), DP-1570 (JX042321), DP-1571 (JX042322), DP-1572 (JX042323), DP-1574 (JX042325) and DP-1575 (JX042326). Four samples were identical with sequences from the *B. divergens/capreoli* cluster. These were DP-1573 (JX042324), DP-1576 (JX042327), DP-1577 (JX042328), DP-1578 (JX042329) and ‘Mia’. As this 18S region in the 3′ end is identical for both of these species, an additional PCR was performed which covered a region of 18S in which discrimination was possible, described as ‘Protocol I’ [19]. Sequences obtained by ‘Protocol II’ [19], revealed that two *Babesia* DP-1576b (JX083979), DP-1578b (JX083982) (as well as the positive control ‘Miab’(JX083981)) were completely identical to *B. divergens.* DP-1573b (JX083983) and DP-1577b (JX083980) were identical to *B. capreoli*. These identifications were possible due to the nucleotides at positions 634 and 666 related to the Genbank sequence FJ944825. The remaining sample DP-1561 (JX042313) was significantly different to the various *Babesia* spp. included into the initial alignment of the 3′ end of 18S. A BLAST search on this variable region alone, revealed a 100% match with an unknown *Babesia* (GU475475) described from red deer (*Cervus elaphus*) in Ireland [19]. The DP-1561 sample was also included in the additional protocol where the 5′region of the end of the 18S was amplified. Analysis of this sequence (Figures 3), DP-1561b (JX083978), revealed similarity to a strain of *Babesia* from Switzerland (also referred to as ‘CH-1’) (DQ312432) and with a Genbank entry assigned as *B. odocoilei*-like sequence from Austria (JN543175); but it did not match any of these sequences completely as it differed by two different nucleotides from each of these sequences.

## Discussion

The realtime-PCR analysis of *I. ricinus* revealed an overall *Babesia* prevalence of 0.9% in our material. This level is comparable to other studies using similar approaches for detection: in Italy the *Babesia* sp. ‘EU1’ prevalence was 0.85% [23], in Estonia 1,4% [24], in Poland 1.6% [25], and corresponds to previous findings in Norway [12,18]. In this study we found evidence of the presence of *B. venatorumB. divergensB. capreoli* and one strain of a *Babesia* very similar to a so far undescribed *Babesia* in questing *I. ricinus*.

The most prevalent *Babesia* in our material was *B. venatorum* found in 71% of the positive ticks (Table 2). In Norway, *B. venatorum* has only been reported from ticks on migratory birds [12]. Although *B. microti* has been previously described from ticks in Norway [10], no ticks with this *Babesia* were detected in the realtime PCR screening followed by confirmation by PCR and sequence analysis. We used primers designed specifically for *B. divergens* group and *in silico* analysis of the target region on 18S rRNA, reveals that there is only marginal complementarity for the realtime PCR primers and probe used in the initial screening in *B. microti* rRNA. Taqman probe is only 71% identical with *B. microti* (AB112050) sequence, whilst forward and rewerse primers are only 77% and 91% identical. Thus it is possible that this species and other related *Babesia* spp. which may vary significantly in this region, may have been missed by our screening analyses. It is also possible that limitations of the use of ammonium hydroxide DNA and incomplete lysis of ticks used in the initial screening may have produced false negative samples. Our prevalence estimate should thereby be an underestimate of the actual *Babesia* spp. prevalence in ticks in Norway.

**Table 2:** *Babesia* species in *I. ricinus* in different locations, and GenBank nucleotide accession numbers of 18S rRNA gene sequences

**Sample ID**	**host**	**Location**	**Genbank ID 5′-end**	**Genbank ID 3′-end**	**Identified as**
DP-1561	*Ixodes ricinus* (n)	21 Tippheia	JX083978	JX042313	*Babesia* spp.
DP-1562	*Ixodes ricinus* (n)	7 Hinnebu	n/a	JX042314	*B. venatorum*
DP-1563	*Ixodes ricinus* (n)	7 Hinnebu	n/a	JX042315	*B. venatorum*
DP-1564	*Ixodes ricinus* (f)	3 Hvasser	n/a	JX042316	*B. venatorum*
DP-1565	*Ixodes ricinus* (n)	1 Mølen	n/a	JX042317	*B. venatorum*
DP-1567	*Ixodes ricinus* (f)	8 Tromøy	n/a	JX042318	*B. venatorum*
DP-1568	*Ixodes ricinus* (f)	5 Risør	n/a	JX042319	*B. venatorum*
DP-1569	*Ixodes ricinus* (f)	8 Tromøy	n/a	JX042320	*B. venatorum*
DP-1570	*Ixodes ricinus* (m)	6 Tvedestrand	n/a	JX042321	*B. venatorum*
DP-1571	*Ixodes ricinus* (m)	6 Tvedestrand	n/a	JX042322	*B. venatorum*
DP-1572	*Ixodes ricinus* (n)	7 Hinnebu	n/a	JX042323	*B. venatorum*
DP-1573	*Ixodes ricinus* (f)	7 Hinnebu	JX083983	JX042324	*B. capreoli*
DP-1574	*Ixodes ricinus* (f)	7 Hinnebu	n/a	JX042325	*B. venatorum*
DP-1575	*Ixodes ricinus* (m)	7 Hinnebu	n/a	JX042326	*B. venatorum*
DP-1576	*Ixodes ricinus* (n)	14 Mundheim	JX083979	JX042327	*B. divergens*
DP-1577	*Ixodes ricinus* (f)	10 Odderøya	JX083980	JX042328	*B. capreoli*
DP-1578	*Ixodes ricinus* (m)	17 Utvik	JX083982	JX042329	*B. divergens*
Mia	Bovine babesiosis	Flekke*	JX083981	n/a	*B. divergens*

n – nymph, f – female, m – male;

n/a - sequencing were not performed.

* -Positive control came from a clinical ill cow sampled from ‘Flekke’ location situated approximately 25 km N of location 15, ‘Brekke’ in Figures 1.

The ticks positive for *Babesia* spp. were recovered from various regions of the country (Figures 1). All ticks infected with *B. venatorum* were found in the southern part of Norway while the *B. divergens* positive ticks were found in the West (Utvik) and South (Odderøya) of Norway. *B. capreoli* was found in south Norway (Hinnebu). The sample identified as the CH1-like strain, was recovered at Tippheia at Hitra (Figures 1). This may indicate some difference in the geographic distribution of the *Babesia* species, with most variants occurring on the South-East coast of Norway. There were a number of sites where we did not find *Babesia* spp. in this study. However, with a prevalence bordering 1%, and with most sites having sample numbers lower than 100, this may be expected. In Surnadal, however, an outbreak of babesiosis in cattle was recorded the year before our sampling, but no positive samples with *Babesia* from ticks from this location were recovered in this study. A study from the southern Norwegian coast [9] found that 17 of 25 cows from Jomfruland were seropositive for *Babesia*. However, no *Babesia* positive ticks were found in Jomfruland in Radzijevskaja *et al*. [18], and this study, when a total of 305 ticks were investigated. More investigations should elaborate on this.

The ticks investigated in this study were questing ticks sampled in the vegetation in spring and early summer. *Babesia venatorum* was encountered more frequently in this study than expected, indicating that there is a widespread distribution of this *Babesia* species on the South-East coast of Norway. *Babesia venatorum* is naturally transmitted by *I. ricinus*[26], and roe deer has been regarded as the main cervid hosts. We do know that the areas sampled on the South East coast of Norway generally has a high roe deer population. The wild cervids are thought to be the main hosts for *I. ricinus* in the areas sampled. As roe deer is regarded as the main vertebrate host for *B. venatorum* it is therefore likely that this *Babesia* can be regarded endemic to Norway. More studies of the vertebrate host population should be carried out to prove this. Detection of host DNA in fed ticks, or additional sampling of wild cervids to identify vertebrate reservoirs in the areas may provide valuable information. Regardless of the infection routes, if *B. venatorum* has similar properties as *B. divergens* which include transovarial and transstadial transmission [27], and may last for two tick generations [28], the infected tick populations may on their own be regarded as reservoirs for *Babesia*[9]. The recording of *B. venatorum* will be of public health importance as these ticks can act as vectors of the pathogen to man in periods of the year filled with outdoor activities. One of the sites of our sampling (Tromøy) is a popular arena for annual festivals with lot of human activity.

This study reports *B. venatorumB. divergensB.capreoli* and the so far undescribed species, *Babesia *CH1-like strain to be present in ticks in Norway. *Babesia ventorum* from ticks on birds and *B. divergens* as a pathogen on cattle has been reported before [12], but this is the first detection of *B. capreoli* in Norway. The vertebrate host of *B. capreoli* is roe deer, and it is closely related to *B. divergens* but is regarded as a separate species [29]. *Babesia capreoli* does not pose a threat to humans or livestock as experiments show that it is not capable of multiplying in blood from humans or cows [29].

In this study we also recovered a special *Babesia* variant from the island of Hitra (DP-1561).

This *Babesia* strain was identical to a red deer (*C. elaphus*) *Babesia* variant found in Ireland [19] when compared to the sequence from the 3′ end of the region of 18S (Figures 2). Zintl and colleagues [19] described the unknown variant they identified using the 3′ end of 18S from red deer. Unfortunately, no sequence information was available for Zintl’s irish isolates at the 5′ end of 18S at the time of analysis. We amplified the 5′ end of DP-1561 and a separate blast search of this sequence revealed that this variant clustered with *Babesia* sp. genotype CH1‘(DQ312432) sampled from red deer in Switzerland [30]. It also clustered with a Genbank sequence from Austria (JN543175), although not part of a published work yet, it was described as a *B*. cf. *odocoilei* from red deer. (DP-1561b differed by only 2 nucleotides with respect to sequence DQ312432 and JN543175. One of the nucleotides in which variation was seen in our sample included a polymorphic site in our sample, representing a G or T, either nucleotides not seen in the Genbank sequences. The remaining altered nucleotide was due to a nucleotide only seen in CH1 (a T nucleotide at position 333), or a nucleotide seen only in *B*. cf. *odocoilei* (a G nucleotide at position 145). The *Babesia* detected in DP-1561 appears to represent a new variant, which seems to be an intermediate version of these two ‘unknown’ genbank entries. This unknown *Babesia* is identical in the 3’ sequence of 18S to the strain that Zintl and colleagues [19] found in Ireland and very similar to what researchers discovered in Switzerland and Austria when comparing the 5′ end of the molecule, hence it should be regarded “CH1-like”. Regardless of its species status Zintl *et al*. [19], Hilpertshauser [30] and the authors of the Genbank entry JN543175 found this variant in red deer. We found this isolate in the West coast of Norway ‘Hitra’, and this island has a very high density of red deer (*C. elaphus*) and roe deer (*Capreolus capreolus*) [31]. Most likely it is the red deer population in Hitra that harbors this *Babesia* recovered in sample DP-1561.

Although screening of wild cervids in Norway may contribute to the knowledge of possible wild reservoirs of these *Babesia* species, it seems from our identified strains that roe deer and red deer may be the most important hosts for the *Babesia* we have found in this study. The possible role of moose and reindeer as reservoirs for *Babesia* in Norway remains unknown. Recently, there have been reports of reindeer fatalities due to *Babesia*[32], and severe infections of reindeer due to *B. divergens* and *B. odocoilei*[33,34], suggesting that reindeer may not be a good reservoir host as these pathogens may give rise to severe disease when encountered. Given that the *I. ricinus* ticks carrying *Babesia* would encounter reindeer babesiosis in reindeer in Norway may be possible. However, as the habitats of roe deer and reindeer rarely overlap and the tick density is relatively low in the mountain habitats of reindeer, this may generally not be the case.

Babesiosis in man and animals normally only occur transiently and symptoms normally are general and mild. It is therefore possible that the disease may be underdiagnosed in Norway. Problems related to the difficulty of diagnosis and the lack of screening laboratories [6], may contribute to a possible under-diagnosis of human babesiosis in Norway.

## Conclusions

This study employed molecular tools for the presence of *Babesia* spp. in questing *I. ricinus* ticks sampled in various regions of Norway. Four species of *Babesia* were encountered suggesting that *Babesia* is distributed in the tick population throughout Southern Norway. *Babesia venatorum* dominated, being identified in 12 out of 17 isolates. *Babesia divergens*, *B. capreoli* and a currently undescribed *Babesia* was detected.

## Endnotes

^a^ The current status of the name *Babesia venatorum* as a formal taxononomic name remains debatable, as full formal species description is not available. These strains have originally been referred to as *Babesia* sp. ‘EU1’ [5]. For simplicity we refer to it as *Babesia venatorum* in the current report.

## Competing interest

The authors declare that they have no competing interest.

## Authors’ contributions

ØØ performed the PCR ,sequencing and molecular analysis of positive ticks from realtime PCR as well as interpretation of the molecular data and preparation of the manuscript.

JR performed the realtime PCR, calculated the prevalence and together with AP collected ticks, and contributed with revision of the manuscript. OR designed the study, contributed with collection of ticks and contributed to the preparation of the manuscript.

All authors read and approved the final manuscript.
